# New quality and quantity indices in science (NewQIS): the study protocol of an international project

**DOI:** 10.1186/1745-6673-4-16

**Published:** 2009-06-26

**Authors:** Beatrix Groneberg-Kloft, Tanja C Fischer, David Quarcoo, Cristian Scutaru

**Affiliations:** 1 Otto-Heubner. Centre, Charité-Universitätsmedizin Berlin, Free University Berlin and Humboldt-University Berlin, Germany; 2 Department of Dermatology and Allergy, Charité-Universitätsmedizin Berlin, Free University Berlin and Humboldt-University Berlin, Germany; 3 Institute of Occupational Medicine, Charité-Universitätsmedizin Berlin, Free University Berlin and Humboldt-University Berlin, Germany

## Abstract

Benchmarking systems are important features for the implementation of efficacy in basic and applied sciences. These systems are urgently needed for many fields of science since there is an imbalance present between funding policies and research evaluation. Here, a new approach is presented with an international study project that uses visualisation techniques for benchmarking processes. The project is entitled New Quality and Quantity Indices in Science (NewQIS). The juxtaposition of classical scientometric tools and novel visualisation techniques can be used to assess quality and quantity in science. In specific, the tools can be used to assess quality and quantity of research activity for distinct areas of science, for single institutions, for countries, for single time periods, or for single scientists. Also, NewQIS may be used to compare different fields, institutions, countries, or scientists for their scientific output. Thus, decision making for funding allocation can be made more transparent. Since governmental bodies that supervise funding policies and allocation processes are often not equipped with an in depth expertise in this area, special attention is given to data visualisation techniques that allow to visualize mapping of research activity and quality.

## Introduction

Progress in science is crucial for the economic development of most countries. This advance is commonly directly related to a country's intramural and extramural governmental and non-governmental funding policy. Funding sources are limited. Currently, the economic crisis leads to a further reduction of the sources and both health care systems and research funding are endangered [1,2]. Therefore, numerous research projects and grant proposals may not be financed [3]. This leads to an enormous potential of arising conflicts. The scientific community discusses these issues in great detail and there is an increasing interest in the underlying processes that lead to the allocation of funding budgets at the international, national and subnational levels [4-6].

## Hypothesis and objectives

We hypothesize that without the use of scientometric techniques, there will be a growing discontent among scientists for funding allocation policies. In this respect, the use of specific benchmarking systems could be of help to implement transparency within funding allocation processes.

While single scientometric and bibliometric methods are known for many areas to dissect research activities of faculties or single scientists, the use of these techniques is often hampered. Reviewing the existing policies in Europe [7] or other general statements [8-11], it becomes clear that we need an improvement in this area. Therefore, the present work describes aims to establish an approach to visualize research quantity and quality indices.

**Figure 1:**
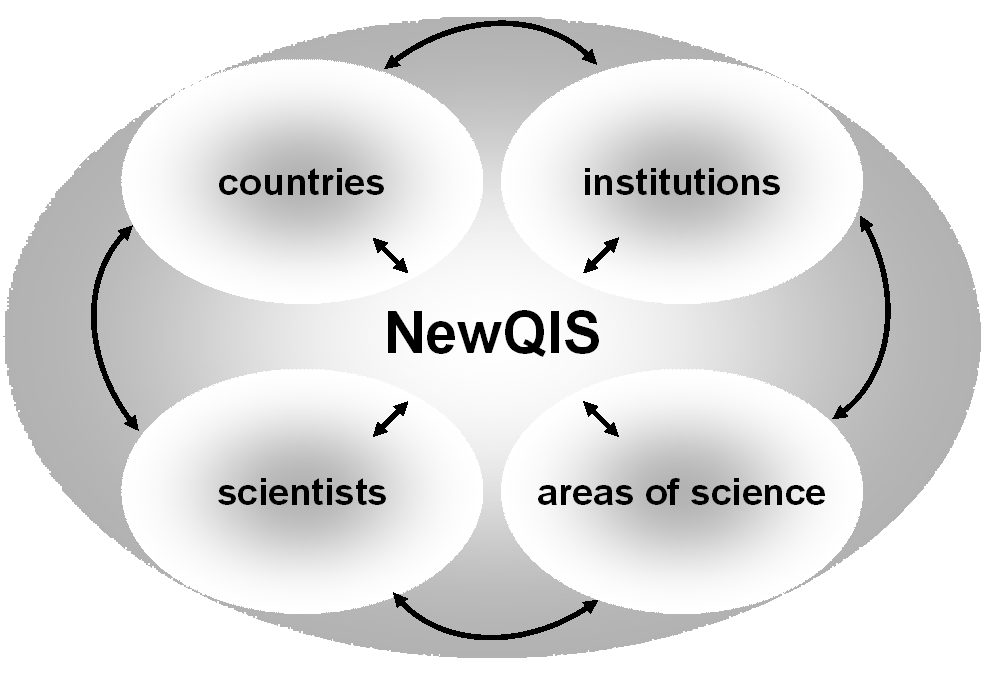
**The NewQIS study protocol can be used to assess research output for 1) distinct areas of science, for 2) institutions, for 3) countries, or for 5) scientists**.

This approach should be usable to assess quality and quantity of research activity i.e. for 1) distinct areas of science, for 2) single institutions, for 3) single countries, for 4) single time periods, or for 5) single scientists (Fig. 1). In addition to this evaluation, NewQIS studies may also be used for comparative issues: In this respect, different fields of science, different institutions, different countries, or different scientists or different time periods can be compared in terms of scientific output. Thus, decision making for funding allocation can also be made more transparent. Our hypothesis is that this approach is a useful tool for all kind of decision making concerning the allocation of funding. The approach can be termed "New Quality and Quantity Indices in Science (NewQQIS)". Arising from this approach, future NewQIS – studies may be conducted to provide a sound basis for the analysis and evaluation of research activity in distinct field of biomedicine.

## Methods

The methods used for the international NewQIS studies are based on classic data bases such as the PubMed, Scopus or the Web of Science. Within these data bases, biomedical research output can be categorized with the numbers of published entries as an index marker for quantity of output. Quantities can be analysed with regard to different main characteristics: 1) specific fields of science, specific organs, diseases or other phenomena 2) countries 3) publication dates 4) authors 5) affiliations depending on the focus of the study. The data can then be transferred to visualisation techniques such as density equalizing calculations.

### Study example

**Figure 2:**
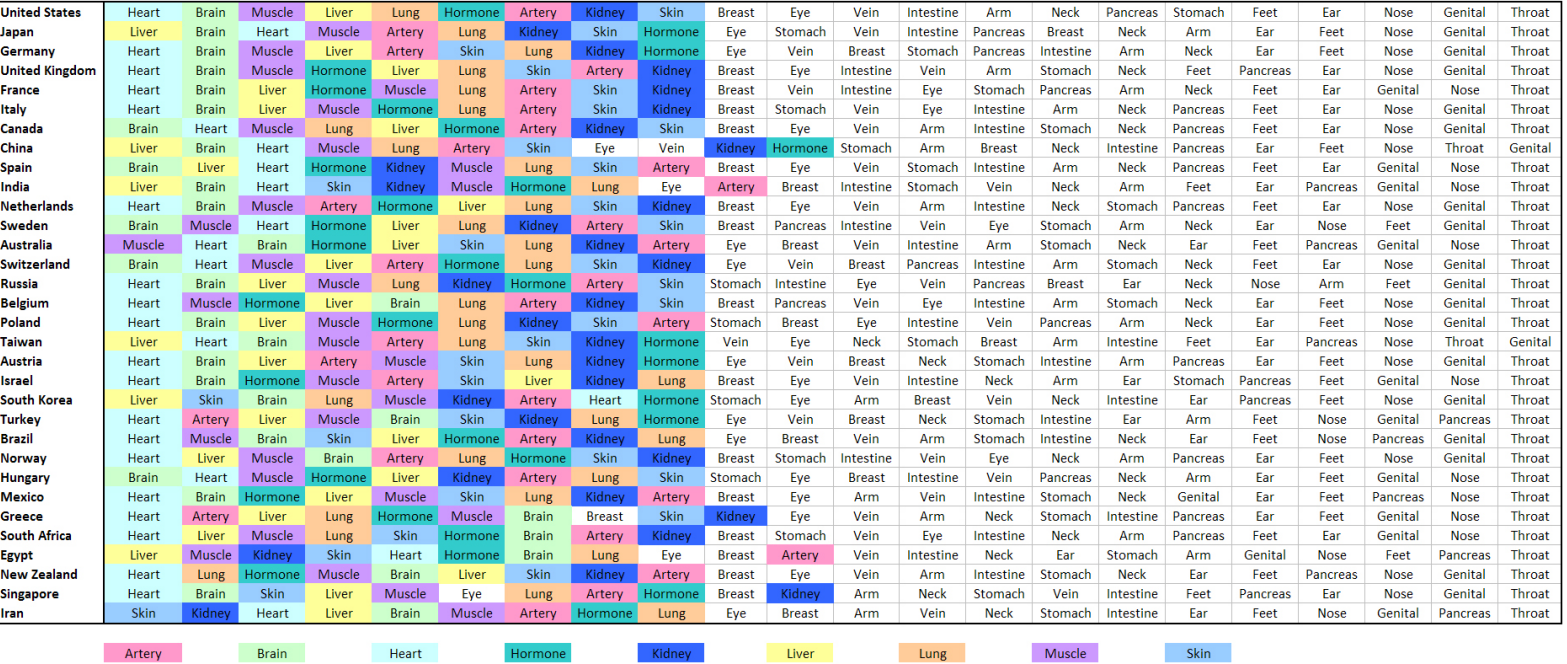
**International differences in focus of research**. Ranking of organs in each country. Scopus data base search. Data from [12] Health Res Policy Syst. 2008; 6: 6. Published online 2008 June 13. doi: 10.1186/1478-4505-6-6. Copyright ^© ^2008 Groneberg-Kloft et al; licensee BioMed Central Ltd.

Three recently published studies may serve as prototype examples for the NewQIS approach and were used to establish the study protocols: Using the two large databases Scopus and Web of Science biomedical research output was categorized with the numbers of published entries as an index marker for quantity of output [12]. The quantities were analyzed with regard to three main characteristics: 1) organs 2) countries 3) publication dates. Density-equalizing mapping was used in this study for the visualization of data. In this respect, the territories were re-sized according to a particular variable, i.e. the number of published items. For the re-sizing procedure the area of each country was scaled in proportion to its total number of published items regarding the organs heart, brain, liver, lung and skin. The specific calculations were based on Gastner and Newman's algorithm [13]. In total, 5,527,558 published items were analysed in this study [12]. Using this approach a dichotomy was shown to be present between Western countries such as the US, UK or Germany and Asian countries such as Japan, China or South Korea concerning research focuses (Fig. 2). This was the first large scale analysis of global research activity and output over the last 50 years and the approach was used to establish the NewQIS protocol.

**Figure 3:**
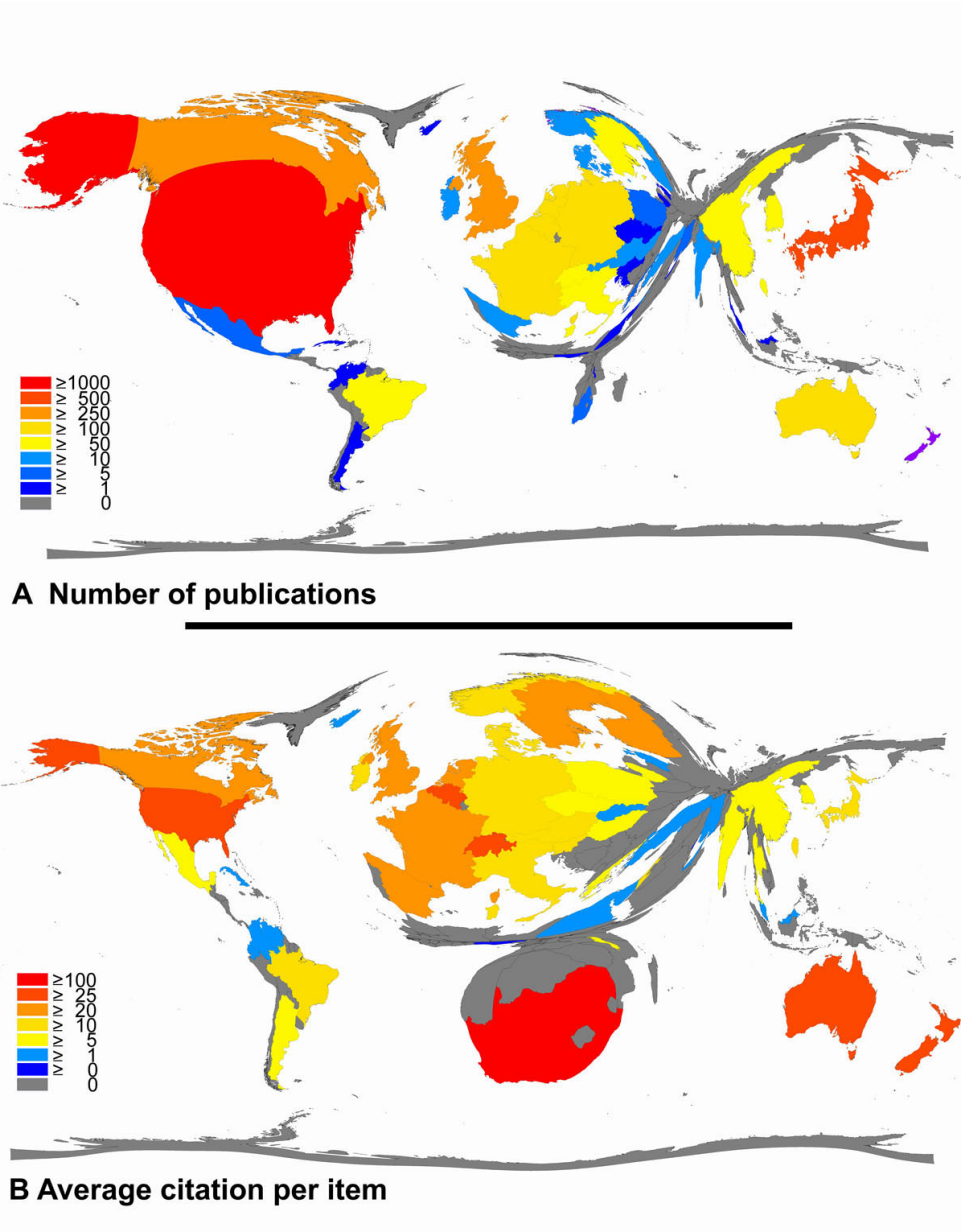
**A. Density-equalizing map illustrating the number of publications in each particular country**. The area of each country was scaled in proportion to its total number of publications regarding animal models of asthma. B. Density-equalizing map showing the average citations per item of each particular country. The area of each country was scaled in proportion to its average number of citations per item regarding animal models of asthma. Data from [14] Börger et al. Journal of Occupational Medicine and Toxicology 2008 3(Suppl 1):S7 doi:10.1186/1745-6673-3-S1-S7 Copyright ^© ^2008 Börger et al; licensee BioMed Central Ltd.

As a second example, the recently published data by Borger et al can be used [14]. Here, different animal models of asthma were analysed using in part the presently proposed NewQIS techniques. Density-equalizing algorithms were used and data was retrieved from the Thomson Institute for Scientific Information database Web of Science. During the period from 1900 to 2006 a number of 3489 filed items were found to be connected to animal models of asthma, the first being published in the year 1968 [14]. These studies were published by 52 countries. The US, Japan and the UK were the most productive countries, participating in 55.8% of all published items (Fig. 3). When analyzing the average citation per item as an indicator for research quality Switzerland ranked first (30.54/item) and New Zealand ranked second for countries with more than 10 published studies [14]. The 10 most productive journals included 4 journals with a main focus allergy and immunology and 4 journals with a main focus on the respiratory system. Two journals had a focus on pharmacology or pharmacy. In all assigned subject categories examined for a relation to animal models of asthma, the field of immunology ranked first. As a last step the numbers of published items were categorized with regard to specific animal species. Here it was found that mice were the preferred species followed by guinea pigs [14]. In summary it was concluded that the use of animal models of asthma is restricted to a relatively small number of countries with major differences in subsets of the analysis. The differences can be attributed to variations in the research focus as assessed by subject category analysis.

In a third example the visualisation procedures were extended to video analysis in order to be able to assess the evolution over the time [15]. In this study, the neighbouring fields of cardiovascular and respiratory medicine served as models for diverging patterns of health research. Density equalizing mapping procedures were used in combination with video analysis. In this study specific areas of major research activity were identified for European countries and in general large differences were found [15]. In this respect, the spatial distribution of published items for cardiac and cardiovascular systems differed in comparison to the distribution for the respiratory system. Large countries dominated the overall number of published items. In order to evaluate the kinetics of publication activity the total publication output of the countries in five years intervals was analysed [15]. A continuing rise of publication numbers was found with a tendency to increased progression after the year 1997. The increase of publications was visualized for the field respiratory medicine using video density-equalizing mapping in one-year steps (see additional file 1).

## Discussion

It is obvious that the policy for the allocation of research grants could benefit from an increase in 1) objectivity and 2) transparency. This is due to the fact that non-transparent and questionable allocation policies have contributed to weakening the reputation of basic and applied research all over the globe. There are numerous studies on research evaluation and policy in countries such as the USA or Australia which have described the existence and nature of this problem in their countries but there is still a lack of approaches that encompass both valid assessment tools and the ability to visualize results. We here establish an international project that uses scientometric tools together with visualising techniques. The NewQIS studies can be efficiently used to dissect scientific progress in closer detail. Here, they may be used to categorize research progress in all fields of science. They may also help for decision making in specific research grant calls. In this case, future NewQIS studies can incorporate parameters such as citation analysis or authorship and institution network analysis. This can be used specifically to evaluate research proposals on a supranational, national or infranational level.

## Conclusion

As a conclusion, the use of the presently described approach to assess research quantity and quality may be used to 1) establish an objective basis for the evaluation of science 2) provide a visualizing platform for non-specialists or non-scientists who coordinate funding and funding policy at governmental or non-governmental levels.

## Competing interests

The authors declare that they have no competing interests.

## Authors' contributions

BGK. TCF, DQ and CS conceived the study protocol, participated in the process of the design of the methodology and drafted and prepared the manuscript. All authors read and approved the final manuscript.

## Supplementary Material

Additional file 1 **Time-space distribution of published items related to respiratory medicine using density-equalizing mapping and one-year steps**. The film sequence visualizes the total increase in respiratory medicine publications. The color coding encodes the total number of published items per country over the time. Data from [15] BMC Health Serv Res. 2009; 9: 16. Published online 2009 January 27. doi: 10.1186/1472-6963-9-16. Copyright ^© ^2009 Groneberg-Kloft et al; licensee BioMed Central Ltd. Click here for file
